# Sparse Detector Imaging Sensor with Two-Class Silhouette Classification

**DOI:** 10.3390/s8127996

**Published:** 2008-12-08

**Authors:** David Russomanno, Srikant Chari, Carl Halford

**Affiliations:** Center for Advanced Sensors, Department of Electrical and Computer Engineering, The University of Memphis, Memphis, TN, U.S.A. 38152; E-Mails: schari@memphis.edu; chalford@memphis.edu

**Keywords:** Electronic fence, imaging sensor, sparse detector array, object identification, Web-service interface

## Abstract

This paper presents the design and test of a simple active near-infrared sparse detector imaging sensor. The prototype of the sensor is novel in that it can capture remarkable silhouettes or profiles of a wide-variety of moving objects, including humans, animals, and vehicles using a sparse detector array comprised of only sixteen sensing elements deployed in a vertical configuration. The prototype sensor was built to collect silhouettes for a variety of objects and to evaluate several algorithms for classifying the data obtained from the sensor into two classes: human versus non-human. Initial tests show that the classification of individually sensed objects into two classes can be achieved with accuracy greater than ninety-nine percent (99%) with a subset of the sixteen detectors using a representative dataset consisting of 512 signatures. The prototype also includes a Webservice interface such that the sensor can be tasked in a network-centric environment. The sensor appears to be a low-cost alternative to traditional, high-resolution focal plane array imaging sensors for some applications. After a power optimization study, appropriate packaging, and testing with more extensive datasets, the sensor may be a good candidate for deployment in vast geographic regions for a myriad of intelligent electronic fence and persistent surveillance applications, including perimeter security scenarios.

## 1. Introduction

By definition, a sparse detector sensor is an imaging device that has a relatively sparse detector array as compared to state-of-the-art imaging sensors. Sparse detector sensors may be a low-cost alternative to traditional, high-resolution imaging sensors, which use dense focal plane arrays, for object classification. Size, cost, and power restrictions preclude the use of traditional imagers in applications that require widespread deployment of the sensors and in scenarios in which the sensors must be regarded as disposable. Classification of sparse detector sensor data is of particular interest when building inexpensive, unattended ground sensors; however, robust classification is challenging due to the paucity of information that can be used to detect and identify various objects.

The design of an unattended ground sparse detector imaging sensor for broad-scale object classification has been prototyped in our laboratory [1-3] with support from the U. S. Army Research Laboratory (ARL). This prototype sensor is being designed and evaluated, in part, to address the need to monitor trails and unimproved roads, which provide routes for drug smuggling traffic, as well as other applications in which broad-scale classification of sensed objects is of high interest [4-5]. Unattended ground sensors that can reliably distinguish between humans and animals are critically needed for several other potential military, homeland security, and commercial applications.

This work complements ongoing research at the Center for Advanced Sensors at the University of Memphis to develop a network of low-cost sensors and intelligent signal processing algorithms that detect and provide a broad-scale classification of humans and vehicles, while ignoring non-utility animals. Since trails and unimproved roads are often the point of entry for illegal aliens, smugglers, and terrorists, effectively monitoring these areas represents a significant challenge to national security. The United States' border with Canada and Mexico is approximately 12,000 km, with many remote and uninhabited sections. Persistent monitoring of these areas is currently not feasible and is restricted to high-traffic areas; however, ubiquitous deployment of sensors to monitor the entire border is of extreme interest. For example, The Department of Homeland Security (DHS) is leading the development of technology for border security via the SBI.net project [6]. This project is attempting to use advanced sensors, such as moving target indication (MTI) radar and thermal infrared cameras, in a network-centric environment, to perform detection, classification, and tracking of humans crossing the border. When fully developed, the estimated cost to deploy this technology along the entire U.S. border is approximately $620K per km (≈ $1 million dollars per mile of border) [7].

Innovative use of alternative low-cost sensors, including imaging sensors, and networking technology may provide a competing or complementary monitoring capability for borders and other application scenarios. For example, suppose a suite of low-cost sensors could be developed that provide a significant percentage of the functionalities offered by the technologies selected for the SBI.net project, but they could only do so for a coverage area of 10 m. If the cost of each sensor was $100 and it costs an equal amount for installation, the total cost would be approximately $20K per km of border. If each sensor had a 10-percent (10%) per year failure rate, there would be an additional recurring cost of $2K per km. Even doubling these costs, there is still a substantial margin between the potential costs of the sensors envisioned as part of our work and the technologies currently under development for comprehensive electronic border security.

The remainder of this paper details the design and evaluation of one approach to a low-cost imaging sensor that has a sparse detector array. It is proposed that such a sensor could be a component of a ubiquitous, low-cost sensor network. Section 2 introduces our sparse detector imaging sensor prototype and an initial motivating application and provides the details of the sensor developed in our laboratory, including the acquisition of images used for subsequent classification. Section 2 also provides details about the Web-services interface and issues for future deployment. Section 3 highlights an initial approach used for classifying the images from the sensor into human and nonhuman classes. Section 4 offers conclusions and future directions.

## 2. Sparse Detector Imaging Sensor

### 2.1. Motivating application

**Figure 1.**
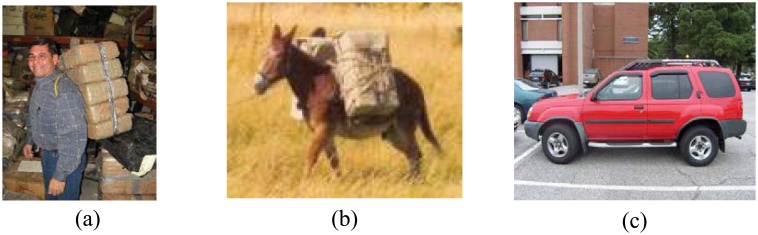
(a) Human with large backpack. (b) Utility animal with packs. (c) Vehicles, such as SUVs.

Typically, smugglers on foot use large packs to transport contraband weighing up to 50 kg along known trails and unimproved roads across the border between the U.S. and Mexico. Smugglers often travel in large groups and the border patrol has inadequate personnel to monitor these vast geographic regions. Therefore, a high degree of confidence in classification algorithms is needed to provide notification when objects of interest are detected to allow authorities to assemble adequate personnel to intercept and apprehend the smugglers along known points on the trails or roads. Moreover, numerous and inexpensive unattended ground sensors are needed for placement at several locations and these sensors should be resilient to false alarms as there is inadequate capacity among the authorities for reacting to false alarms. In our initial application domain, such trails and unimproved roads are abundant, but most of the routes are known. However, the routes have many travelers that are not of interest, including non-utility animals and humans that do not fit the profile of interest. In typical deployments, a sensor would be placed near a trail having a width of approximately 1 to 1.5 m or near an unimproved road of width of approximately 3 to 5 m. These sensors can be located where vegetation or other environmental features can be used to hide placement. The trails and roads are located in areas that are considered open range. Wild horses, cattle, deer, large cats, dogs, rabbits, and pigs are just a few of the non-utility animals that use the same trails as humans moving through the area. Figure 1 illustrates a variety of object types for which object identification is of high priority.

### 2.2. Sparse Detector Model

**Figure 2.**
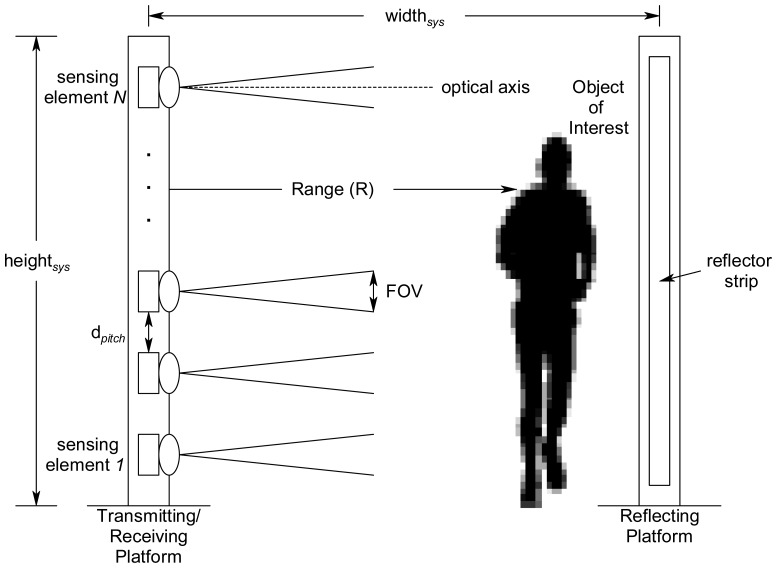
Sparse detector system model configuration.

Our prototype sparse detector imaging sensor system can be viewed as a near-infrared implementation of the model described by our colleagues at the Center for Advanced Sensors as shown in Figure 2. As modeled in Robinson *et al.* [8], the sparse detector system consists of an array of sensing elements deployed in a vertical configuration on a transmitting/receiving platform. The sensor system also has a reflecting platform that is placed at a distance represented by *width_sys_*. Each sensing element has a detector and a dedicated collecting lens component. Each sensing element is arranged such that its optical axis is perpendicular to the plane of the vertical array comprised of *N* sensing elements that are placed at a distance of *d_pitch_* apart. The range to the object of interest along the optical axis is represented by *R*. The field of view (FOV) of each sensing element is calculated as a function of the detector area and optics of each sensing element. The overall height of the sensor system is represented by *height_sys_*.

Robinson *et al.* [8] have developed and used this model for rough trade-off analyses, which include the effects of the optics, atmosphere, detectors, object-of-interest characteristics, and system characteristics, such as detector pitch and normalized detectivity. As further described in [8], the sensor can be classified as either having a staring or a scanned system type [9]. Since it consists of a stationary sparse detector array with no device for scanning the image across the detectors it could be viewed as a staring system in which the vertical resolution is dependent upon the deployment of the individual sensing elements, which comprise the overall device. However, it can also be regarding as a scanning system since the image will effectively be ‘scanned’ across the detectors by the object-of-interest's motion in which the horizontal sample rate of the system will be determined by the integration time of the detector.

### 2.3. Sensing elements

**Figure 3.**
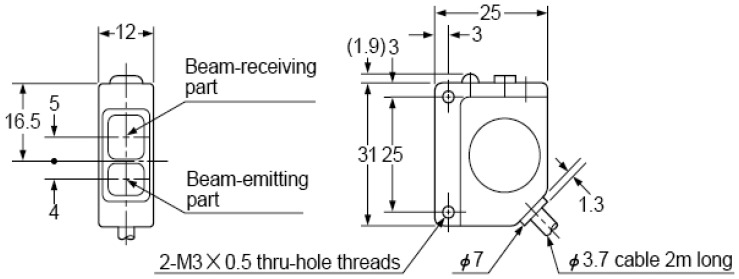
CX-RVMS retro-reflective photoelectric sensing element (all units mm) [10].

The CX-RVMS retro-reflective infrared sensor [10], as shown in Figure 3, was used as the principal sensing element to construct the prototype sensor system to obtain signature data in a laboratory and controlled-field environment for further analysis. The CX-RVMS was selected because of its suitability for both laboratory and controlled-field testing. It has environmental reliability, which includes BSi IP67 waterproof construction, and it is vibration resistant with its interior fully filled with resin. Moreover, this sensing element has a 1 ms or less response time, 5 m sensing range, and can operate from -25 to 60 °C. An alternative, lower-power sensing element would be required for wide-scale deployment; however, the CX-RVMS served our purposes for proof of concept and to acquire initial signature data.

### 2.4 Sensor system assembly

**Figure 4.**
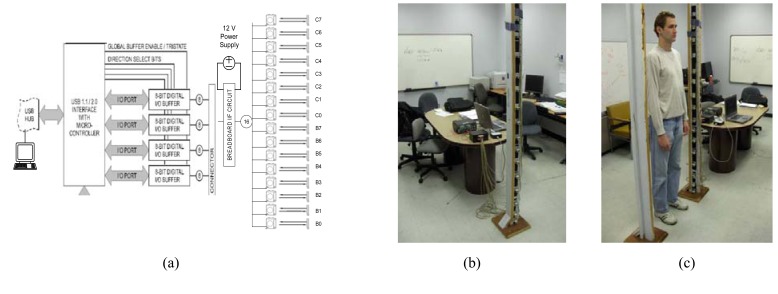
(a) Prototype sensor logic diagram. (b) Transmitting/receiving platform. (c) Human walking between transmitting/receiving and reflecting platforms.

The prototype sparse detector sensor was assembled by placing 16 CX-RVMS retro-reflective sensing elements at 12.7-cm intervals in a vertical configuration (*N*=16, *d_pitch_* = 12.7 cm, and *height_sys_* ≈ 2.2 m with respect to the model in Figure 2). Each sensing element can be regarded as an optical trip wire and was attached to a supporting platform with reflectors mounted on an opposing platform to provide the break-beam curtain. Each CX-RVMS sensing element was interfaced to a USB-DIO-32 digital input board using a simple voltage divider breadboard circuit to provide the required 0 to 5V output. The input board was subsequently connected to a host computer via a USB interface. Figure 4(a) illustrates the configuration in which sensing element B0 (the first sensing element) is placed at 17.7 cm from the platform base and sensing element C7 (the sixteenth sensing element) is placed approximately 208.2 cm from the platform base. Figure 4(b) is a photograph of the transmitting/receiving platform with the sensing elements interfaced to a host computer in the laboratory environment and Figure 4(c) illustrates a human walking between the platforms with *width_sys_* ≈ 1.2 m.

### 2.5. Data acquisition

The driver software for the digital input board interfaced to the sensor uses a 32-bit DLL compatible with any Windows programming language. A C/C++ program was developed to acquire signature data assuming at least one sensing element's beam would be broken as an object traverses through the sensor's optical curtain for initial testing purposes. Therefore, data acquisition started when a break-beam event first occurred and continued at a sampling rate of approximately 10 ms until none of the sensing elements detected a break in their beam.

**Figure 5.**
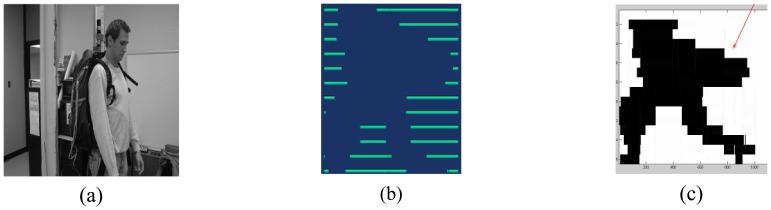
(a) Human with backpack. (b) Break-beam pattern from the sensor for a human wearing a backpack. (c) Silhouette generated from the break-beam pattern acquired by the sensor.

A raw dataset is created by the C/C++ program by writing a single ASCII file for each object detection event as a string of 1s and 0s corresponding to no-break and break, respectively, for sensing element B0 through C7 as the object passes through the sensor's optical curtain. Each of the 16 sensing elements is sampled in parallel, which provides data for a ‘16 × *I*’ matrix. Variable ‘*I*’ is the number of samples taken for each sensing element and will be constant within a single file; that is, the same number of samples will be taken for sensing elements B0 through C7 for a given object's data acquisition. However, ‘*I*’ will vary among files as it depends on the specific object and the interval of time in which at least one sensing element detected a break-beam event, that is, ‘*I*’ depends on the speed, size, configuration, and other variables of the object. Figure 5(b) is a visualization of the optical trip wires for a typical break-beam pattern for a human with a small backpack and was produced using the Visualization Toolkit (VTK) [11].

**Figure 6.**
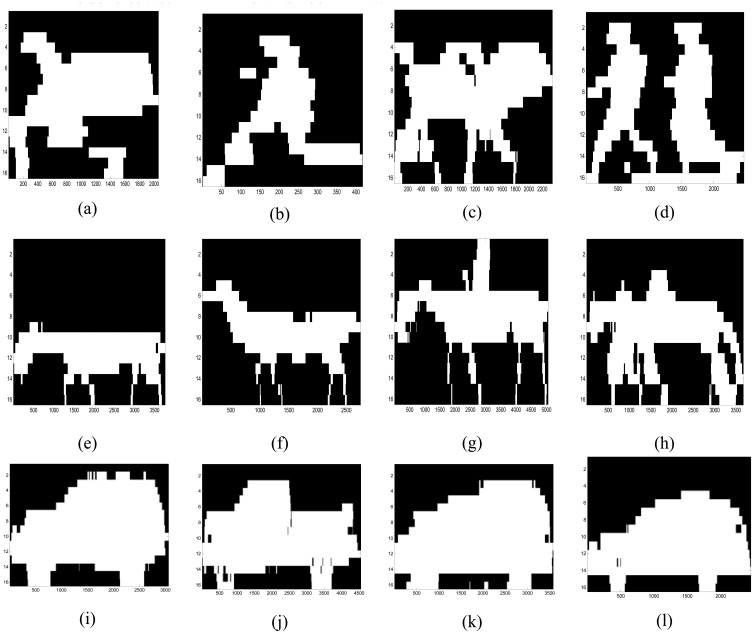
Silhouettes created from break-beam patterns acquired from the sparse detector imaging sensor: (a) human with large backpack. (b) human without backpack. (c) two humans with large backpacks. (d) two humans without backpacks. (e) donkey. (f) llama. (g) horse with human rider. (h) horse with pack led by human. (i) sport-utility vehicle (SUV). (j) pick-up truck. (k) van. (l) car. The x-axis denotes the number of samples and the y-axis denotes a sensing element number (1-16).

Figure 5(c) illustrates a representative ‘crude image’ or silhouette that was created from the break-beam pattern acquired from the prototype sensor using a MATLAB program. For the testing and evaluation of the sensor and the human versus non-human classification task using silhouettes described later in this paper, a total of 512 datasets were acquired from the sensor, consisting of 137 animals, 226 humans, and 148 vehicle-type signatures. Figure 6 presents representative silhouettes for a variety of objects that were acquired from the sensor, including: (a) human with large backpack; (b) human without backpack; (c) two humans with large backpacks; (d) two humans without backpacks; (e) donkey; (f) llama; (g) horse with human rider; (h) horse with backpack led by human; (i) sportutility vehicle (SUV); (j) pickup truck; (k) van; and a (l) car.

For each image in Figure 6, the x-axis lists the number of samples taken to generate the silhouette and the y-axis lists a sensing element number (1-16). Note that the sensing element order on the y-axis of each image is reversed from the order used to configure the sensor in Figure 2. The maximum value of the x-axis provides a relative indicator of the speed and the width of the object or objects in the silhouette since wide objects would break the optical curtain for a longer time than narrow objects. The x-axis maximum value can also be used to compare the relative speed of similar object types, that is, a human running would result in fewer samples acquired by the sensor as compared to a similarly sized human walking through the optical curtain.

These images or silhouettes are remarkable given that they were produced from a sensor with only 16 detectors placed at 12.7-cm intervals. The data was collected with the sensor configured with *width_sys_* ranging from approximately 1 to 5 m (that is, the distance between the transmitting/receiving and reflecting platforms). In the case of multiple objects traversing between the sensor's transmitting/receiving and reflecting platforms, as in Figures 6(c), (d), (g) and (h), our data acquisition program required a continuous break of at least one optical trip wire (sensing element) to acquire the data for both objects in one dataset file. Multiple objects were not used as training datasets for the initial classification algorithms reported later in this paper. A number of samples were acquired for each object type as appropriate, for example, different strides, postures, orientations, speed, etc.

### 2.6. Service-oriented software interface

In conjunction with the silhouette collection and classification algorithm development, we have been developing software for the prototype profiling sensor to facilitate integration within a network-centric environment using service-oriented computing [12] infrastructure and Web services. Network access to the sensor has also been developed and has been exposed via Web Services Description Language (WSDL) so that client applications can be designed for the profiling sensor using the development environment of the programmer's choice. All sensor data and embedded operations, such as self-test, sensor sample rate, alarm thresholds, and other configuration parameters and functionality are being exposed as Web services using WSDL [3]. Such a service-oriented architecture with Internet Protocol (IP) networking provides a framework for including the sensor in a variety of intelligent monitoring applications, deployment, and interoperability within existing frameworks. For example, an application may need to task a given deployment of the profiling sensor to detect any object that it senses. This could be accomplished via the *detect_object* service call, which requires the client to specify the time interval for which the sensor should report if at least one optical beam was broken.

**Figure 7.**
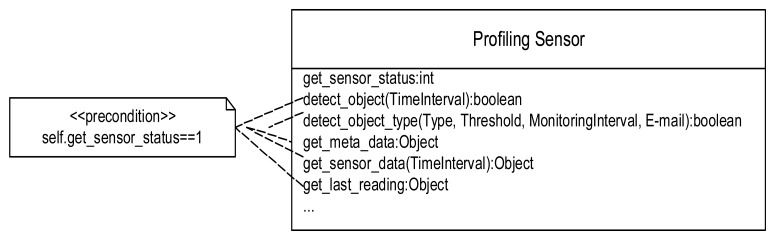
Example Web services for the profiling sensor described with UML.

More sophisticated services, such as *detect_object_type*, require the client to specify the object type to detect (for example, human, human_large_backpack, SUV, horse, generic object, etc.), the threshold (e.g., an integer specifying the number of occurrences of the specified object to detect during the specified time interval), the time interval for monitoring for the specified object, and an e-mail address to notify the recipient of the detection or identification event that satisfies the specified constraints. Example Web services for the profiling sensor are represented in Figure 7 using the Unified Modeling Language (UML). Note that the majority of the service calls require the sensor to have completed a successful self test to ensure the sensor is aligned and operating correctly, which is represented as a UML pre-condition constraint in Figure 7, before the client can successful invoke the other services.

**Figure 8.**
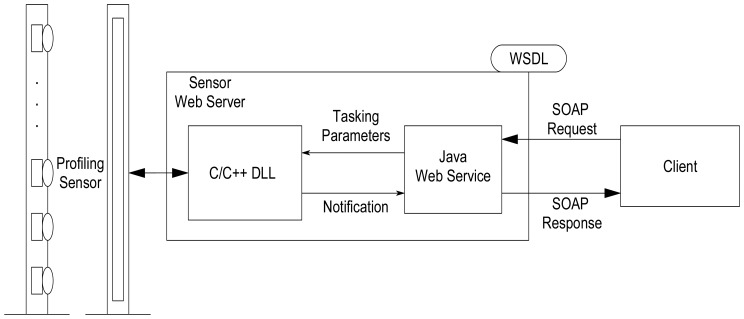
Profiling sensor service-oriented interface to client.

Currently, the prototype version of the sensor provides a WSDL file that serves as a wrapper for a Java program that implements the Web service and subsequently invokes the lower-level sensor API developed in C/C++ as shown in Figure 8. The WDSL and the associated Extensible Markup Language (XML) schema describe all types, methods, arguments, and responses of the sensor. The client maps the abstracted types and structures specified by the WSDL file to the specific bindings required by the client's host programming language. The client communicates with the sensor via the Java Web service using Simple Object Access Protocol (SOAP) calls, which are exchanges of XML-based messages over a network. Using this approach, the developer can use the software implementation platform of their choice when using the profile sensor in custom applications without the burden of knowing the lower-level sensor application programmer's interface (API) or device drivers, which is typically required when using sensors and other devices in custom applications.

Light-weight versions of the profiling sensor are planned that are bundled with an embedded processor. Such devices will have a very limited computational platform powered via batteries, wireless interface, and an environmentally hardened construction. These devices will be evaluated to determine the level of computational complexity supported, including the feasibility of hosting a full-featured, Web-services interface as is provided by the initial prototype.

To accommodate ubiquitous deployment, data communication protocols must be carefully considered. Minimizing wireless communications saves both bandwidth and power. The break-beam patterns of each sensor are highly compressible, so one approach is to transmit the collected data over a wireless communication channel to a base station each time the sensor is activated as an alternative to designing the sensor to include an embedded processor to locally classify the data and to process various tasking parameters.

Power requirements are also a major concern in the design of the sensor, as long battery life is highly desirable. The choice of detectors affects power consumption along with the choice of data communication protocols. Transmission of high-level image classifications consumes less power than the transmission of the raw data, even if it is compressed. Naturally, the decision regarding the centralized base station approach versus the distributed architecture, with the intelligence on the sensor, will be determined by the specific application.

### 2.7 Other deployment issues

**Figure 9.**
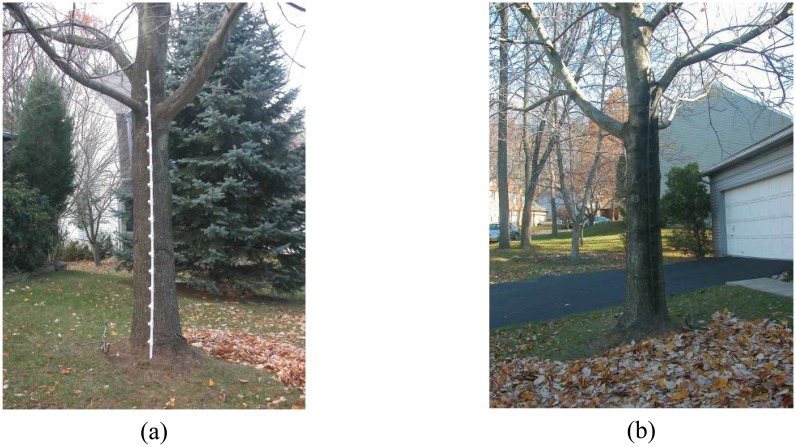
Example camouflage of a sparse detector sensor: (a) Profiling sensor realized with sensing elements in PVC pipe and transmitting/receiving platform mounted on a tree. (b) Profiling sensor realized with sensing elements in PVC pipe with transmitting/receiving platform painted to blend in with a tree [1].

Concealment of the device is also a requirement in many applications. To be effective, a smuggler or person crossing a border illegally must not be aware of the location of the sensors. Figure 9 demonstrates a simple means of camouflaging a notional packaging of a profiling sensor using paint. Another means of hiding the device that has been proposed is to distribute the sensing elements horizontally along a path. Each beam-break device would operate at both a different height and a different distance along the path. By distributing the sensing elements, the profiling sensor system is less conspicuous. By keeping track of the location of each sensing element, the profile can be constructed by synchronizing the data from the distributed beam-break sensors. The distributed approach requires additional computational overhead for coordination, which may be processed either locally or at a centralized base station.

While the sensing elements described in this paper are an active near-infrared emitter/detector, it would be possible to construct a profiling sensor from passive infrared detectors. Klett et al. [5] describe the analysis of optical and radiometric calculations that are necessary to begin evaluating a passive infrared sparse sensor system. These types of sensors are often used in motion detectors to activate outdoor lighting and domestic security systems. Lenses could be designed to provide a sufficiently narrow field of view to capture one element of a profile. This type of detector has the advantage of not requiring an active light source or a reflector to operate since the radiation originates from the object-of-interest. In addition, a long wave infrared (LWIR) version, in which the majority of the sensed energy is emissive rather than reflective, may minimize false alarms, which are common with commercial passive infrared detectors [4]. Also, a detailed power analysis and techniques to decrease the power consumption of sparse detector sensors and their associated communications package is an additional opportunity for research.

## 3. Classification Algorithms

Five algorithms were tested to classify data obtained from the profiling sensor into two classes: human and non-human. The algorithms were the Naïve Bayesian Classifier (NB) [13], Naïve Bayesian with Linear Discriminant Analysis for dimensionality reduction (NB + LDA) [14-15], *K*-Nearest Neighbor classifier (*K*-NN) [16], Soft Linear Vector Quantization (SLVQ) [17], and Support Vector Machines (SVM) [18]. Although we report profiling sensor classification results in prior work [1-2], those results were not for the two-class problem, nor were those algorithms tested with the extensive 512 datasets described in Section 2.5. For completeness, a review of each algorithm is discussed before presenting the latest classification results.

### 3.1. Naïve Bayesian classifier

A Naïve Bayesian classifier assigns a test sample to a class with the highest posterior probability among the *J* classes, with the assumption that each feature of the sample is statistically independent [13]. The posterior probability of the *j*th class, *P*(*ω_j_*|*x*), is the probability of a class given the data sample (vector) *x* = [*x_1_*,* x_2_*,…,*x_M_*], where *x_i_* is the *i*th feature of the data vector. Bayes' theorem relates the posterior probability to prior probability of the *j*th class, *P*(*ω_j_*); class conditional probability, *P*(*x*|*ω_j_*); and the total probability of *x* or evidence, *P*(*x*) as shown in (1).

(1) $$P(\omega_{j}|x)=\frac{P(\omega_{j})P(x|\omega_{j})}{P(x)}$$

The prior probability *P*(*ω_j_*) is given by $P\left(\omega_{j}\right)=\frac{n_{j}}{N}$, where *n_j_* is the number of data samples belonging to the *j*th class and *N* is the total number of samples. The total probability of *x*, *P*(*x*), is shown in (2):

(2) $$P(x)=\sum_{j=1}^{J}P(\omega_{j})P(x|\omega_{j})$$

If each class is expected to occur with equal probability, then classification based on posterior probability depends only on the class conditional probability. The calculation of the class conditional probability is commonly performed using the parametric approach, where a probability distribution model, such as the Gaussian distribution, is assumed for the data points. The parameters for the distribution (e.g., mean and standard deviation for Gaussian) are then estimated from the training samples. Thus, the class conditional probability of the *j*th class given a data vector can be expressed as shown in (3):

(3) $$P(x|\omega_{j})=P(x_{1}|\omega_{j})P(x_{2}|\omega_{j})\ldots P(x_{M}|\omega_{j})=\left(\frac{1}{\sigma_{1,j}\sqrt{2\pi }}\exp\left(\frac{x_{1}-{\hat{\mu }}_{1,j}}{2{\hat{\sigma }}_{1,j}^{2}}\right)\right)\left(\frac{1}{\sigma_{2,j}\sqrt{2\pi }}\exp\left(\frac{x_{2}-{\hat{\mu }}_{2,j}}{2{\hat{\sigma }}_{2,j}^{2}}\right)\right)\cdots \left(\frac{1}{\sigma_{M,j}\sqrt{2\pi }}\exp\left(\frac{x_{M}-{\hat{\mu }}_{M,j}}{2{\hat{\sigma }}_{M,j}^{2}}\right)\right)$$

In Equation (3), (*μ̂_k_*_,_*_j_*, *σ̂_k_*_,_*_j_*)represent the estimated mean and standard deviation of *k*th feature corresponding to the *j*th class. With the assumption of equal prior probability for each class for a test sample vector *xtest*, if *P*(*xtest*|*ω_j_*) has the highest value among all class conditional probabilities, then the test sample is assigned to the *j*th class.

### 3.2 Naïve Bayesian classifier with Linear Discriminant Analysis for dimensionality reduction

Dimensionality reduction involves projecting a variable from an *M*-dimensional to an *S*-dimensional space, such that *M* > *S*. Linear Discriminant Analysis (LDA) is a dimensionality reduction technique in which the data is projected onto a lower dimension so that the overlap between the classes to be discriminated is minimized making it popular as a preprocessing technique for the Naïve Bayesian approach. In LDA, each axis in the new space is a linear combination of all the axes in the original space. In matrix notation, the LDA transformation can be expressed as in (4):

(4) $$Y=W^{T}X$$

In Equation (4), *Y* is the new space with *S* dimensions, *X* is the original space with *M* dimensions, and *W* is the transformation matrix. The LDA algorithm finds a transformation matrix that maximizes the Fisher criterion [14] as a function of *W* and is shown in (5):

(5) $$F(W)=\left|\frac{W^{T}S_{B}W}{W^{T}S_{W}W}\right|$$

For *C* number of classes, the between class scatter matrix [15], *S_B_*, is shown in (6), in which *P_i_* and *M_i_* are the probability of occurrence and mean of the *i*th class, while $M_{0}=\sum_{i=1}^{c}P_{i}M_{i}$ the within class scatter matrix [15], *S_W_*, is shown in (7), in which Σ*_i_* is the covariance matrix of the *i*th class:

(6) $$S_{B}=\sum_{i=1}^{c}P_{i}(M_{i}-M_{0}){\left(M_{i}-M_{0}\right)}^{T}$$

(7) $$S_{W}=\sum_{i=1}^{c}P_{i}\sum_{i}$$

Also, *S_B_*_,_*_Y_*=*W^T^S_B_*_,_*_X_W* and *S_W_*_,_*_Y_* = *W^T^S_W_*_,_*_X_W*, in which *S_B_*_,_*_Y_* and *S_W_*_,_*_Y_* are the between and within class scatter matrices of *Y*, while *S_B_*_,_*_X_* and *S_W_*_,_*_X_* are the between and within class scatter matrices of *X*. The matrix *W* that maximizes *F* is the matrix of eigenvectors of $\frac{S_{B}}{S_{W}}$.

To project the data onto the lower-dimensional space with *S*-dimensions, the eigenvectors corresponding to the *S* most significant eigenvalues are used for the transformation. Once the data is projected onto this lower dimensional space, the *S* features are then used by the Naïve Bayesian classifier for classification of the data.

### 3.3. K-Nearest Neighbor classifier

The *K* Nearest Neighbor classifier assigns a test sample to the *j*th class if a majority of its *K* nearest neighbors, which are from the training data, belong to the *j*th class [16]. The training and testing samples are defined in an *M*-multi-dimensional space. The neighborhood is defined using a distance measure, such as the Euclidean distance, in which the distance between a test sample, *xtest*, and any training sample *x* is given by (8):

(8) $$\text{dist}(\text{xtest},x)=\sqrt{\sum_{i=1}^{M}{\left({\text{xtest}}_{i}-x_{i}\right)}^{2}}$$

The *dist*(*xtest*,*x*) is computed between *xtest* and every stored data sample (training data). The training samples are then sorted in ascending order according to the distances from the test sample. The first *K* training samples are then picked and the test sample is assigned to the class to which most of the *K* samples belong.

### 3.4. Soft Learning Vector Quantization

Learning Vector Quantization is a technique to learn prototypes of various classes in Nearest Prototype Classifiers (NPC). Unlike the *K*-NN classifier, in which the distance between the test sample and all training samples is computed, with NPCs, the distance between the test sample, *xtest*, and prototype vectors for each class is computed. The test sample is assigned to a class whose prototype has the least distance from the test sample. Prototypes represent each class in an *M*-dimensional space. One or more prototypes can be used to represent a given class to help accommodate variations within a class. If the set {*θ_i_*, *ω_i_*} represents the prototype vectors and the corresponding classes, *i*= 1, 2, …, *N*, in which *N* is the number of prototypes, then, *ω_i_* is the class of the *i*th prototype with *ω_i_* taking values from 1, 2, …, *C*, in which *C* is the number of classes. The NPC approach finds *p* as shown in (9) in which *dist*() is a distance measure such as the Euclidean distance. The test sample is then classified to class *ω_p_*.

(9) $$p=\underset{i}{\arg\min}(\text{dist}(\text{xtest},\theta_{i}))$$

There are many versions of LVQ, such as LVQ1, LVQ2.1, LVQ3, OLVQ1, and OLVQ3 [19]. Each version differs in the way the prototypes are updated in the learning process. For example, in version LVQ2.1, first, the prototypes for the various classes are initialized. In each iteration, for a training data point, two prototypes, *θ_A_*, *θ_B_*, are picked based on the Euclidean distance from the training data point. The two prototypes are not updated if both belong to the same class as the training data point. If *θ_A_* belongs to the same class as the training sample, and *θ_B_* does not, then the prototypes are updated as shown in (10):

(10) $$\begin{array}{c} \;\theta_{A}(t+1)=\theta_{A}(t)+\alpha (t)(x-\theta_{A}(t))\\ \theta_{B}(t+1)=\theta_{B}(t)-\alpha (t)(x-\theta_{B}(t)) \end{array}$$

Another condition on the update is that the test point *x* should lie close to classification boundary; therefore, in the previous case, $\frac{\text{dist}(x,\theta_{A})}{\text{dist}(x,\theta_{B})}>Th$, where *Th* is a threshold.

Seo *et al.* [17] introduced the Soft Learning Vector Quantization (SLVQ) technique in which the prototypes are represented by Gaussian mixtures. The prototype representing the *j*th class is represented by the *j*th component of the Gaussian mixtures. In other words, it is assumed that a particular data point belonging to the *j*th class is generated by the *j*th component of the Gaussian mixture. While LVQ produce hard decisions, SLVQ generates soft decisions. SLVQ provides degrees of membership for a test sample with respect to the various classes. Since the prototypes are components of a Gaussian mixture, the *j*th prototype is described by its parameters: mean and standard deviation, *θ_j_* = {*μ*,*σ*}. Let *S* = {*x*,*y*} represent the set of all training data points, *x*, and corresponding class labels, *y*, while *T* = {*θ*, *ω*} represents the set of prototypes, *θ*, and the classes they represent, *ω*. The prototypes *θ* are calculated using gradient descent while optimizing the cost function in (11):

(11) $$\log L=\frac{\sum_{i=1}^{N}p(x,y|T)}{\sum_{i=1}^{N}p(x,y|T)+p(x,\bar{y}|T)}$$

In Equation (11), *p*(*x*, *y* |*T*) is the probability of a data point *x* being generated by a prototype of the correct class and *p*(*x*, *y̅* |*T*) is the probability of the data point being generated by a prototype of the incorrect class.

### 3.5. Support Vector Machines

Support Vector Machines (SVMs) are classifiers that address the two-class problem by identifying a separating hyper-plane that leaves the maximum margin from each of the two classes [18]. The hyperplane is defined by *h(w)* = *w^T^x*+*w*_0_, in which *w* and w_0_ characterize the direction and position of the hyper-plane, respectively. Since neither of the classes is to be given preference, the hyper-plane should be located equidistant from the nearest points from class 1 and class 2. The distance of any point *x* from the hyper-plane is given by (12). Further, *w* and *w_0_* can be scaled so that the distance of the hyper-plane from the nearest points in class 1 and class 2 is set to unity. The margin is then given by (13). Further, if *x* belongs to class 1, then *w^T^x*+*w*_0_ ≥ 1 and if *x* belongs to class 2, then *w^T^x*+*w*_0_ ≤ 1.

(12) $$\frac{g(x)}{\left\|w\right\|}$$

(13) $$\frac{1}{\left\|w\right\|}-\left(\frac{-1}{\left\|w\right\|}\right)=\frac{1}{\left\|w\right\|}+\frac{1}{\left\|w\right\|}=\frac{2}{\left\|w\right\|}$$

The goal is to maximize the margin $\frac{2}{\left\|w\right\|}$ between the nearest points of class 1 and class 2 when the classes are separable. SVM finds *w* and w_0_ by minimizing the cost function (14) under the constraints: *y_i_*(*w^T^x_i_*+*w*_0_)≥1 where *y_i_* = 1 if the training data point *x_i_* belongs to class 1, and *y_i_* = 1 if the training data point *x_i_* belongs to class 2.

(14) $$J(w)=\frac{1}{2}{\left\|w\right\|}^{2}$$

In the case when the two classes are not separable, the optimization is more complicated, and SVM finds *w*, *w_0_*, and *ξ* by minimizing the cost function [18] shown in (15) under the constraints *y_i_*(*w^T^x_i_*+*w*_0_)≥1−*ξ_i_*, where *i*= 1,2, …,*N* in which *N* is the number of training samples and *ξ_i_* ≥ 0.

(15) $$J(w)=\frac{1}{2}{\left\|w\right\|}^{2}+C\sum_{i=1}^{N}\xi_{i}$$

### 3.6. Classification of the prototype profiling sensor data

The majority of the algorithms were implemented using MATLAB and the data was classified off line. The SLVQ technique was implemented using the RSLVQ toolbox [17]. However, the low-level profiling sensor software developed in C/C++ has been designed to easily accommodate the ‘plug-and-play’ of a variety of algorithms to support real-time classification. As described in detail in Section 2.5, as an object passes through the sensor's optical curtain, a 16 × *I* matrix is generated. Each row corresponds to the output of a sensing element. For each of the 16 sensing elements, when the break-beam event occurs, the samples are recorded as 0s; otherwise, the samples are recorded as 1s. The number of 0s for each sensing element was used as features. Thus, for each object, 16 features were measured. Furthermore, to make the features independent of the speed of an object, each feature was normalized between 0 and 1 by dividing each feature value by the feature with the highest value for that object. The overall goal was to classify an object as human or non-human. Vehicles and animals formed the non-human class, but if these two classes were grouped into a single class, the variance of the non-human class would become very large. To overcome this problem, class 1 represented humans, class 2 represented animals, and class 3 represented vehicles. An object was classified as non-human if it belonged to class 2 or class 3. Since the SVM technique is devised for two-class problems, when SVM is used as a classifier, the one-against-one strategy with majority voting is used to address the three-class problem. Table 1 summarizes the classification rate (CR) for the data using the five classification algorithms previously described.

The classification results indicate that SVM performed the best using the sample datasets, closely followed by NB, NB + LDA, *K*-NN, and SLVQ, respectively. Errors in the classification rate are also influenced by the feature extraction process. The feature extraction process used here did not account for the details of the structures in the silhouette. For example, for animals, the fact that some have four legs and that they are in pairs, with one pair in the front and one pair in the rear of the animal was not accounted for by the feature extraction process. This becomes a critical issue in differentiating a human carrying a large backpack from a medium-sized animal, especially when each object is captured in a single silhouette. Future research in the area of profiling sensor algorithm development will include adding rules in the feature extraction process to accommodate structural information in silhouettes and the capability to identify and classify multiple objects of the same or different classes captured in the same image as in Figures 6(c), (d), (g) and (h).

### 3.7. Impact of sensing element configuration on the classification rate

After the baseline classification rate (CR) for human versus non-humans was established using the sensor assembly described in Section 2.4 for the algorithms described in Sections 3.1 through 3.5, further analysis was performed to establish additional baseline results. These results document how the number of sensing elements and their arrangement impacted the CR with respect to the human versus non-human classification task using the 512 datasets previously described.

**Figure 10.**
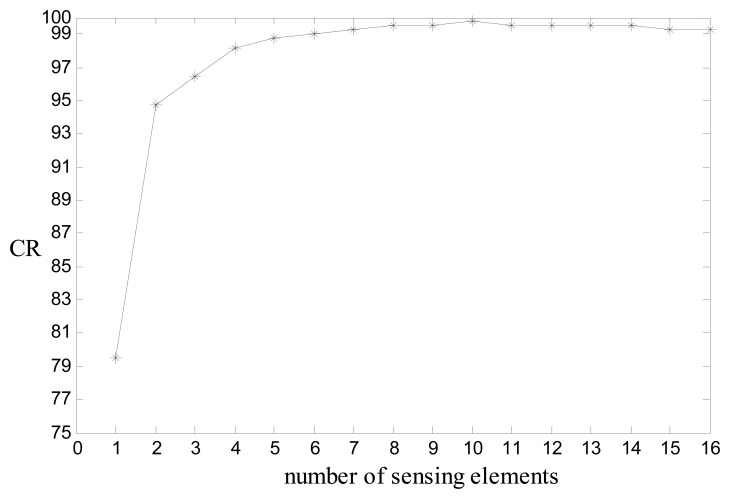
Highest CR(%) for the human versus non-human classification task obtained using the SVM classification algorithm for the subsets containing 1 through 16 sensing elements.

The CR was determined for all five algorithms using every subset of the set containing the 16 sensing elements *s* = {1, 2 … 16} configured as described in Section 2.4. The highest CR obtained for each subset of sensing elements was recorded. The results reported in Figure 10 and Table 2 are for the SVM algorithm. The combination of sensing elements that provided the highest classification rate for a particular subset of *s* is shown in Table 2.

The results indicate that when using the SVM algorithm on the 512 datasets, CRs over 90% for the human versus non-human classification task can be achieved using only two sensing elements (element 4 and 11, with respect to Figure 2). The best CR of 99.7% was achieved using 10 sensing elements. The improved performance using 10 sensing elements rather than 16 can be explained as follows. There were 16 sensing elements in the nominal sensor system, each of which can be thought of as a dimension in a 16-dimensional space. When an object passes through the optical curtain, a data point in this multi-dimensional space is generated. Classification algorithms, such as SVM, classify data points in multi-dimensional space by establishing boundaries between classes. Dimensions corresponding to certain sensing elements can be irrelevant for differentiating between classes and in fact may be responsible for increased overlap between data points of different classes. Such a scenario results in a decrease in class separability and increases classification errors. Thus, when outputs from such sensing elements are ignored in the process of classification, the CR can be increased, depending upon the specific classification task. Also, it was observed that the highest CRs for each subset of *s* was obtained by the SVM algorithm except for the case of a subset containing only 3 elements, in which the NB technique performed the best with a CR of 96.7%, using sensing elements 1, 4, and 11.

It must be emphasized that this analysis was performed to establish baseline results using a nominal sparse detector configuration with a representative dataset containing 512 object signatures of humans, animals, and vehicles. Testing with additional algorithms, including the fusion of multiple algorithms, is warranted using more extensive and varied datasets, as well as testing modifications to the profiling sensor system assembly parameters, such as *d_pitch_* or sampling rate. Lastly, additional analysis of other classification tasks, such as human with no backpack versus human with a backpack, SUV versus car, horse versus deer, etc., is needed to provide a more comprehensive study of the classification rate as a function of the number of detectors, their arrangement, and the specific classification task.

## 4. Conclusions

A simple, active near-infrared prototype imaging sensor has been presented to show the feasibility of using a sparse detector array for capturing and subsequently classifying naïve images or silhouettes of objects. The sensor appears to be a low-cost approach for discriminating among humans and non-humans in unattended ground deployments. Such an approach to designing an imaging sensor may be critical for wide-scale deployments in which the sensor is considered disposable; therefore, the cost of the sensor is crucial. An initial set of algorithms were evaluated using 512 datasets of humans, animals, and vehicles with respect to their ability to classify the data acquired from the sensor into human versus non-human classes. Empirical analysis using these datasets shows that over ninety-nine percent (99%) accuracy is feasible in categorizing individually sensed objects into specific classes of interest. Although not included as part of the results summarized in this paper, the sensor and associated algorithms appear to be resilient to false alarms induced from non-human signatures; however, more detailed analysis is required to determine ‘false alarm’ probabilities.

In addition, the sensor's low-level device driver software has been designed so that various classification algorithms can be inserted and objects can be classified in real-time. The software API has been wrapped to facilitate integration into a service-oriented architecture by providing a WSDL interface to the high-level sensor functionality.

Future directions are being pursued along a variety of focus areas, including acquiring more signature data from the prototype sensor for a wider variety of objects typically found in open range. Also, a more detailed algorithm analysis that relates the resolution of the sensor to its object discrimination capabilities is needed to determine the optimal number of detectors and their arrangement within the sensor system for a given classification task.

In many applications of the sensor, it will be imperative to execute the detection and classification algorithms using a light-weight, low-cost computing platform embedded within the sensor. Minimizing network bandwidth by transmitting only high-level classification results, as opposed to raw or compressed data or silhouettes is desirable in many potential applications. However, other applications are anticipated in which a human will be in the loop to directly classify the sensed data; therefore, human perception studies to evaluate the efficacy of manual classification of the naïve images or silhouettes is also warranted.

A power consumption analysis and optimization effort is needed prior to deployment, as well as developing techniques for camouflage or covert placement. Development of a second version of the prototype sensor, which will be more durable to support further field testing in typical application scenarios, is planned. Ultimately, the realization of the prototype sparse detector sensor concept as a ‘sensor on a stick’ with a self-contained power supply and an embedded system for light-weight computation and communication capability within a network-centric, service-oriented environment is a worthwhile goal.

## Figures and Tables

**Table 1.** CR(%) for human and non-human objects using 16 sensing elements.

**NB**	**NB+LDA**	***K*-NN**	**SLVQ**	**SVM**
97.4	96.7	96.5	95.1	99.2

**Table 2.** Sensing element combination yielding the highest CR (SVM algorithm).

**Number of sensing elements in the subset of*s*(cardinality of*s*)**	**Sensing elements in the subset of*s*(order as in** Figure 2**)**	**CR (%)**
1	13	79.4
2	4, 11	94.7
3	4, 10-11	96.4
4	4, 10-12	98.1
5	4, 5, 10-12	98.7
6	1, 4, 10 - 12, 15	99.0
7	2, 4, 8, 11-13, 15	99.2
8	1, 2, 4, 8, 11-13, 15	99.5
9	1-4, 8, 11-13, 16	99.5
10	1, 3, 4-7, 11-14	99.7
11	1-8, 11-13	99.5
12	1-9, 11-13	99.5
13	1-13	99.5
14	1-13, 15	99.5
15	1-15	99.2
16	1-16	99.2
